# IL-1 Vaccination Is Suitable for Treating Inflammatory Diseases

**DOI:** 10.3389/fphar.2017.00006

**Published:** 2017-01-31

**Authors:** Eric Assier, Natacha Bessis, Jean-François Zagury, Marie-Christophe Boissier

**Affiliations:** ^1^UMR 1125 Institut National de la Santé et de la Recherche MédicaleBobigny, France; ^2^Sorbonne Paris Cité Université Paris 13Bobigny, France; ^3^EA 4627, CNAMParis, France; ^4^Assistance Publique–Hôpitaux de Paris, HUPSSD, Service de RhumatologieBobigny, France

**Keywords:** IL-1, vaccination, inflammation, chronic disease, cytokines

## Relevance of vaccination

Anti-cytokine therapy associated with immunosuppressive drugs has demonstrated high efficiency in treatment of several autoimmune diseases, including rheumatoid arthritis (RA). Current treatments approved for use in patients are targeting inflammatory cytokines TNF-α, IL-1, or IL-6 and more drugs are undergoing clinical evaluation (Semerano et al., 2016a).

Three types of cytokine inhibitors had been developed in RA. The great majority of them is constituted by monoclonal antibodies (mAbs) directed against the cytokine or its receptor. A second category is based on recombinant proteins combining soluble receptors of cytokine stabilized by the Fc domain of IgG1 antibodies. A third type of anti-cytokine drugs is constituted by recombinant receptor antagonist that binds to receptor without induction of signaling.

One common feature of these inhibitors is their high interaction with the cytokine or its receptor that could induce excessive inhibition and some drawback for development. A common side effect is the production, after treatment, of anti-drug antibodies that result in frequent primary or secondary resistance to the treatment. In addition all these drugs have limited effect in the majority of cases; for instance in rheumatoid arthritis, targeted treatments induce 6-month remission in 25–30% patients, and a response different of remission in 50% cases. Partial resistance represents, in the first 6 months, 70% of patients. Furthermore, available anti-cytokine drugs are expensive and need frequent long-term administration.

In this context, a novel type of anti-cytokine drugs based on vaccination is emerging (Semerano et al., 2012). In this case, therapeutic antibodies are produced by the individual itself. This approach has the advantage to generate antibodies that are well-tolerated because of the absence of xenogenic epitopes.

The saga of anti-TNF-α vaccination recapitulates the different steps of such strategy. Several vaccination approaches were conducted in parallel to develop a vaccine against TNF-α. In order to induce a B cell response, DNA vaccination, introduction of a foreign Th cell epitope and coupling TNF-α (or peptides of TNF-α) with carrier proteins were developed in animal models of RA.

A vaccine (TNF-K) constituted by coupling human TNF-α to the carrier protein KLH (keyhole limpet hemocyanin) was studied extensively in animal models of arthritis and in several clinical trials. TNF-K vaccine induced the generation of anti-human TNF-α antibodies that didn't cross react with mouse TNF-α. Transgenic mice expressing human TNF-α (TTG mice) were a suitable model of arthritis to evaluate the efficacy of TNF-K vaccine. In this model, TNF-K vaccine resulted in the production of anti-TNF-α neutralizing antibodies that protected mice against established arthritis (Le Buanec et al., 2006). Anti-TNF-α antibody production was reversible and could not be stimulated by TNF-α administration, as this approach did not induce any cellular-mediated immunity against TNF-α. Therapeutic antibody production was not altered by methotrexate co-administration and the protective effect was correlated with the levels of anti-TNF-α antibody production in sera of vaccinated mice. TNF-K efficacy was compared to infliximab in TTG mice and showed a delay before arthritis inhibition linked to the induction of therapeutic antibodies. This delay did not induce significant alterations of the paws versus infliximab-treated mice, and could not be reduced by simultaneous administration of infliximab in mice during TNF-K vaccination. Indeed, co-administration of TNF-K and infliximab led to a lower response to the vaccine, probably due to epitope masking: TNF-K being constituted with the full human cytokine, infliximab could recognize TNF-α, and increase the clearance of the vaccine. Experiments in animal models of *Listeria monocytogenes* and *Mycobacterium tuberculosis* recently shown that mice vaccinated with a TNF conjugate did not develop a hypersensitivity to these infections (Assier et al., 2016). A first clinical trial (phase IIa) was conducted with RA patients who previously experienced secondary failure of TNF-α inhibitors. Several doses of TNF-K and different schedules of administration were used in order to give rise to therapeutic anti-TNF-α antibodies (Durez et al., 2014). TNF-K vaccine was well tolerated and this first trial held promising clinical improvements. However, a second clinical trial (phase IIB/II) conducted with a larger panel of RA patients did not reach significant therapeutic benefits. This failure could be due to the absence of detection of neutralizing anti-TNF-α antibodies in sera of patients.

Similarly to anti-TNF studies, several anti-cytokine vaccination approaches were developed in autoimmune models against different cytokines including IL-1β (Table 1). These vaccines were composed either with entire cytokine or peptide of cytokine, linked to various carrier proteins (KLH, VLP, Ova, DTT). The use of the full cytokine to compose a vaccine presents some limitations. The cost of recombinant purified cytokines is high and their use induces a polyclonal antibody response against all exposed epitopes. Among these antibodies, only a variable proportion will exert neutralizing capacities and there is a potential risk of unwanted reaction against a shared epitope.

**Table 1:** **Applications of anti-cytokine vaccinations in autoimmune diseases**.

**Cytokine target**	**Product**	**Diseases**	**Animal species**	**References**	**Clinical trial**
mIL-1β	Mouse IL-1β peptides/KLH	Arthritis	Mouse	Bertin-Maghit et al., 2005	Pre-clinical
mIL-1 (α and β)	Mouse IL-1α/IL-1β/VLP	Arthritis	Mouse	Spohn et al., 2008	Pre-clinical
hIL-1β and mIL-1β	Human and murine IL-1β/VLP	Type 2 Diabetes	Mouse	Spohn et al., 2014	Pre-clinical
hIL-1β	Human IL-1β/VLP	Type 2 Diabetes	Human	Cavelti-Weder et al., 2016	Phase I
mIL-6	Modified mouse IL-6	Arthritis, MS	Mouse	Galle et al., 2007	Pre-clinical
	Mouse IL-6 peptide/KLH	SSc	Mouse	Desallais et al., 2014	Pre-clinical
	Mouse IL-6 peptide/KLH	DTH	Monkey	Desallais et al., 2016	Pre-clinical
hIL-15	Modified human IL-15	Arthritis	Monkey	Rodríguez-Álvarez et al., 2016	Pre-clinical
mIL-17A	Mouse IL-17A/Ova	MS	Mouse	Uyttenhove and Van Snick, 2006	Pre-clinical
	Mouse IL-17A/VLP	Arthritis, MS	Mouse	Röhn et al., 2006	Pre-clinical
	Mouse IL-17A/VLP	Autoimmune Myocarditis	Mouse	Sonderegger et al., 2006	Pre-clinical
mIL-18	Mouse IL-18 plasmid	SLE	Mouse	Bossù et al., 2003	Pre-clinical
mIL-23	Mouse IL-23p19 peptide/KLH	Arthritis	Mouse	Ratsimandresy et al., 2011	Pre-clinical
	Mouse IL-23p19 peptide/HBc Ag	Chronic Colitis	Mouse	Guan et al., 2013	Pre-clinical
hIFN-α	Human IFN-α/KLH	SLE	Hum. Transg. Mouse	Zagury et al., 2009	Pre-clinical
	Human IFN-α/KLH	SLE	Hum. Transg. Mouse	Mathian et al., 2011	Pre-clinical
	Human IFN-α/KLH	SLE	Human	Lauwerys et al., 2013	Phase I/II
	Human IFN-α/KLH	SLE	Human	Ducreux et al., 2016	Phase I/II
hTNF-α	Human TNF-α/KLH	Arthritis	Hum. Transg. Mouse	Le Buanec et al., 2006	Pre-clinical
		Arthritis	Hum. Transg. Mouse	Delavallée et al., 2008	Pre-clinical
		Arthritis	Hum. Transg. Mouse	Biton et al., 2011	Pre-clinical
		Arthritis	Mouse	Assier et al., 2012	Pre-clinical
		Arthritis	Hum. Transg. Mouse	Semerano et al., 2013	Pre-clinical
		Arthritis	Human	Durez et al., 2014	Phase IIa
mTNF-α	Modified mouse TNF-α	Arthritis, Cachexia	Mouse	Dalum et al., 1999	Pre-clinical
	Human TNF-α plasmid	Arthritis	Mouse	Shen et al., 2007	Pre-clinical
	Mouse TNF-α peptide/VLP	Arthritis	Mouse	Chackerian et al., 2001	Pre-clinical
	Mouse TNF-α/TNF-α peptide/VLP	Arthritis, Infections	Mouse	Spohn et al., 2007	Pre-clinical
	Mouse TNF-α peptide/KLH	Septic shock	Mouse	Capini et al., 2004	Pre-clinical
	Mouse TNF-α peptide/KLH	Arthritis	Mouse	Sun et al., 2016	Pre-clinical
	Mouse TNF-α peptide/DTT	Arthritis	Mouse	Zhang et al., 2016	Pre-clinical
rTNF-α	Rat TNF-α plasmid	Arthritis	Rat	Wildbaum et al., 2000	Pre-clinical
mVEGF-A	Mouse VEGF/VEGF peptide/KLH	Arthritis	Mouse	Semerano et al., 2016b	Pre-clinical

*DTH, delayed-type hypersensitivity; DTT, transmembrane domain of diphtheria toxin; MS, multiple sclerosis; HBc Ag, hepatitis B core antigen; KLH, keyhole limpet hemocyanin; SLE, systemic lupus erythematosus; SSc, systemic sclerosis; VLP, virus-like particles*.

The use of peptide of cytokine allows limiting antibody generation to selected epitopes. In return, the conformation and the peptidic sequence of the cytokine that interact with the receptor have to be determined. Peptides are often selected in interacting zone of cytokine with its receptor in order to block this interaction. Beyond this highest potential selectivity, the cost of peptides is lower and could lead to similar protections than its entire cytokine counterpart. In this sense, VEGF neutralization by both vaccinal approaches was studied in the collagen-induced arthritis (CIA) model. Vaccines were either constituted by the full length mouse VEGF-A or a peptide (Vpep1) selected in the sequence of VEGF-A, linked to the carrier protein KLH. Vpep1 was chosen for its potential interaction with the VEGF-A co-receptor Neuropilin-1, implied in pathologic angiogenesis. Both types of VEGF vaccines led to the production of anti-VEGF polyclonal neutralizing antibodies. Clinical and histological scores of inflammation and destruction were reduced as well as synovial vascularization (Semerano et al., 2016b). Thus, restraining antibody response to a single peptide sequence with a peptide vaccine could protect immunized mice from arthritis.

The use of peptides of cytokine could be also an asset when cytokine are composed of two chains that could be shared with other cytokines. A vaccinal approach against IL-23 was conducted with peptides chosen in the IL-23p19 subunit. IL-23 is important for the generation of Th17 lymphocytes that are implied in autoimmune diseases. IL-23 is constituted by the IL-12p40 subunit, shared with IL-12, and the specific IL-23p19 subunit. Because IL-12 and IL-23 could have opposite effects in autoimmune models, it was of great importance to inhibit selectively IL-23. In this context, two different teams have conducted experiments in CIA and colitis models. The first vaccine constituted by IL-23p19 peptides linked to KLH was protective in CIA model, whereas the second constituted by IL-23p19 peptides fused to hepatitis B core antigen was protective in colitis.

## Targeting IL-1 in chronic diseases

IL-1 is a major inflammatory cytokine that act at the systemic and local levels (Cavalli and Dinarello, 2015). IL-1 is encoded by two distinct genes giving rise to two related but functionally distinct proteins: IL-1α and IL-1β that interact with two IL-1 receptors. IL-1R2, as a decoy receptor, binds to IL-1 but does not transmit a signal. Signaling is assumed by IL-1R1 that is expressed on the surface of most cell types. IL-1α and IL-1β are primarily expressed as precursors. IL-1α precursor could be release from necrotic cells in a fully active form. Thus, IL-1α acts as an alarmin and could induce sterile inflammation. By contrast, IL-1β precursor is inactive and need to be cleaved to become active. Several enzymes are implied, as proteinase-3 and elastase from neutrophils or caspase-1 from hematopoietic cells. In other cells, caspase-1 exists as a pro-enzyme that needs to be cleaved by a macromolecular complex named the inflammasome. NLRP3 (also known as cryopyrin) is one of the major component of inflammasome. Mutations leading to a gain of function of NLRP3 protein are associated with high amounts of IL-1β secretion and autoinflammatory diseases.

In brief, IL-1α is expressed locally, whereas IL-1β is expressed at the systemic level and in inflamed sites as synovial fluids in rheumatoid arthritis or gouty arthritis (McInnes and Schett, 2007; Richette and Bardin, 2010). IL-1α and IL-1β binding could be limited by the naturally occurring inhibitor IL-1 receptor antagonist (IL-1Ra). IL-1Ra can bind to IL-1 receptor with greater affinity than either IL-1α or IL-1β. A recombinant version of IL-1Ra (anakinra) was developed, but its therapeutic use is limited by its short-lived effect leading to frequent injections to the patient. Two other IL-1 blockers were developed and present lower clearance rates. Rilonacept is a soluble decoy receptor that inhibits primarily IL-1β, but also IL-1α, and canakinumab is a monoclonal antibody that neutralizes specifically IL-1β.

It was suggested that specific targeting of IL-1β, may be beneficial over systemic IL-1 blockade. As an example, canakinumab was effective for the treatment of acute gouty arthritis, whereas rilonacept was not. In animal models, several studies targeting IL-1α and IL-1β by a vaccinal approach reinforce this hypothesis. Vaccines were obtained by linking mouse or human IL-1 cytokines to Virus Like Particles (VLPs). These compounds led to high production of anti-IL-1α or anti-IL-1β neutralizing antibodies by vaccinated mice. In the CIA model, both type of vaccines conduct to a higher protection against arthritis than daily administration of mouse IL-1Ra. In a second model of arthritis, the Collagen Antibody-Induced Arthritis model (CAIA), immunization with the IL-1β vaccine protects strongly against arthritis, whereas anti-IL-1α vaccine has no effect (Spohn et al., 2008). IL-1β vaccine was shown to be also effective in a type 2 diabetes model, and its human counterpart was well tolerated in a phase I clinical trial in patients with type 2 diabetes (Cavelti-Weder et al., 2016). In another study, IL-1β was specifically targeted by vaccines constituted by peptides of IL-1β linked to KLH. Peptide sequences were chosen in regions interacting with the receptor and led to the generation of neutralizing anti-IL-1β antibodies. One IL-1β vaccine showed a protective effect in the CIA model on clinical and histological signs of inflammation (Bertin-Maghit et al., 2005). Thus, selective inhibition of IL-1β gave promising results and could be achieved with a peptide-based vaccine. However, targeting IL-1 or its receptor remains an unresolved issue, with contrasting animal and *in vitro* data arguing in favor of either specific cytokine or receptor blockade, but no conclusion that is relevant to the clinical use of these agents.

## Candidates for a vaccine targeting IL-1

The best candidates are chronic diseases with flares, IL-1 dependent, and showing some improvement in previous clinical trials with currently used anti-IL-1 treatments. The low cost of development could allow treatments of patients in Southern countries, where access to healthcare is a concern for most of people. Research on vaccination targeting IL-1β is still ongoing. Preclinical studies with a peptide-based vaccine targeting IL-1β are currently focused on carrier protein and adjuvant, and clinical studies are planned. The use of vaccination targeting IL-1 extends now from initial rheumatic diseases, such as RA and crystal-induced arthritis (such as gout or acute chondrocalcinosis), to autoinflammatory diseases, such as systemic juvenile idiopathic arthritis (JIA), cryopyrin-associated periodic syndromes (CAPS), and familial Mediterranean fever (FMF). IL-1 inhibitors are also promising in two highly prevalent inflammatory diseases, encountered as co-morbidities in patients with rheumatic diseases, namely arteriosclerosis and type 2 diabetes.

## Author contributions

EA and MB draft the manuscript. NB and JZ revised the manuscript.

### Conflict of interest statement

EA and NB have no disclosure. JZ is shareholder of the company Peptinov. MB and JZ have received a Grant from the French Ministries of Research and Industries (FUI) for the development of anti-IL1 peptide vaccination.
